# From narrative to machine-readable logic: Formalising and validating local indigenous knowledge for rural early warning systems

**DOI:** 10.4102/jamba.v18i1.2036

**Published:** 2026-05-05

**Authors:** Asti Amalia Nur Fajrillah, Rudy Hartanto, Lukito Nugroho

**Affiliations:** 1 Department of Electrical and Information Engineering, Faculty of Engineering, Universitas Gadjah Mada, Yogyakarta, Indonesia; 2 Department of Information Systems, School of Industrial Engineering, Telkom University, Bandung, Indonesia

**Keywords:** local indigenous knowledge, early warning systems, socio-technical systems, rural resilience, information and communication technology for development

## Abstract

Technological-based early warning systems (EWS) in rural Indonesia have shown limited long-term adoption because centralised, top-down mechanisms fail to incorporate the contextual triggers trusted by local communities, resulting in a disconnect in how warnings are understood, leading to inappropriate responses. Field evidence reinforces this problem; the usage of Information and Communication Technology (ICT)-based disaster information systems remained below three-quarters of users and the perceived benefits were also limited. At the same time, Local Indigenous Knowledge (LIK) has long played a critical role in disaster preparedness in rural communities. Local Indigenous Knowledge demonstrated universal adoption and was consistently considered more useful in preventing loss of life, fishing gear, catch and boats. Although prior studies have attempted to integrate LIK into disaster technologies, existing frameworks rely heavily on expert-driven knowledge extraction from qualitative interviews. This approach produces expert systems with unvalidated rules, limiting their credibility and scalability. To address this gap, this study proposes a socio-technical integration framework that systematically incorporates LIK into EWS. The framework was developed through a qualitative inquiry involving 17 in-depth interviews with fishermen and a focus group discussion (FGD) with eight fishermen community leaders from three coastal provinces of Indonesia, and its contextual foundation was further validated using 438 fishermen survey across the same regions.

**Contribution:**

The framework introduces three stages: (1) LIK acquisition from community experience, (2) validation through empirical community consensus and scientific explanation and (3) structured integration into EWS. This staged community validation approach reduces dependence on tacit expert judgement and supports future integration with data-supported decision processes.

## Introduction

Natural disasters, particularly hydrometeorological hazards such as floods, droughts and storms, remain a significant threat in Indonesia. In 2023, these hazards accounted for 99.35% of all recorded disasters, according to the National Agency for Disaster Countermeasure of Indonesia (BNPB). With over 83 971 villages spread across the country, most of Indonesia’s population resides in rural areas, of which approximately 63.12% are classified as disaster-prone regions. Beyond structural exposure, rural vulnerability is also shaped by non-structural factors such as low digital literacy, weak system trust and poor contextual relevance of disaster information (Andersson et al. 2020; Chahinian et al. 2023; Sakalasuriya et al. 2020). These challenges are further complicated by the limited adoption of technology-based early warning systems (EWS) (Andersson et al. 2020; Sakalasuriya et al. 2020). In a rural context, an effective EWS is not merely a technical achievement but a matter of contextual integration; successful implementation depends on how well the technology accommodates human aspects. It requires a seamless coupling between technological infrastructure and human decision-making processes. Within this perspective, technology must adapt to the user’s environment to ensure that the automated signals are successfully translated into appropriate action. Top-down EWS often fail because they provide generic, centralised warnings that lack this essential coupling, specifically omitting the observable environmental triggers and action anticipation cues inherent in Local Indigenous Knowledge (LIK). This mismatch leads to a breakdown in interpretability, resulting in low trust and delayed mitigation decisions (Boas et al. 2020; Chisty et al. 2021; Hammood et al. 2020; Perera et al. 2020). Over time, this misalignment threatens the sustainability of EWS implementation (Andersson et al. 2020; Sakalasuriya et al. 2020; Sufri et al. 2020). Findings from a survey of 438 fishermen confirm this design-reality gap, while fewer than three-quarters of fishermen used ICT-based platforms, LIK remains universally used and trusted. From an Information System (IS) perspective, disaster technologies must be designed by prioritising the human aspect, ensuring that technical components align with local practices and values. Within this framework, LIK serves as the essential interpretive bridge, providing the logic required for communities to trust and act upon technical warnings. In rural settings, bottom-up approaches that encourage community participation and local innovation have proven more effective and sustainable than externally driven solutions (Cai, Li & Cheng 2023; Jayanthi et al. 2022; Susanti et al. 2023). In recent years, the smart village concept has gained increasing attention as a development approach that empowers communities to utilise local strengths while adopting appropriate technologies (Dembovska et al. 2023; Jayanthi et al. 2022). Rather than replacing local practices, the smart village approach integrates technology with existing knowledge systems, demonstrating how ICT can support resilience when adapted to local needs and social structures (Alfiah & Koesoemawati 2024; Defe & Matsa 2024; Jayanthi et al. 2022). This alignment makes the smart village concept a relevant foundation for strengthening disaster preparedness in rural coastal communities. Despite the acknowledged potential of LIK as local practices that become innovative solutions for community resilience, existing research has largely focused on documentation rather than establishing a structured process for technological integration (Hiwasaki et al. 2014; Limpo et al. 2022; Lin & Chang 2020; Wang et al. 2019). Most frameworks rely on subjective expert interpretation and lack a community-consensus validation stage, which limits their scalability and credibility (Akanbi & Masinde 2018). Furthermore, prior research has not explained how LIK can be transformed into structured decision rules compatible with digital platforms. Accordingly, there is a need for a systematic process to acquire, validate and integrate LIK into technology-based EWS. This study addresses this gap by answering the following research questions:

***RQ1:*** *How can tacit, narrative-based local indigenous knowledge (LIK) from coastal fishing communities be systematically transformed into a formalised, machine-readable format for disaster warning systems?*

***RQ2:*** *How can community validation be used to empirically evaluate the reliability and actionable utility of the formalised LIK rules?*

This research focuses on the design and evaluation of the underlying logic to provide a computational foundation that bridges human observation and automated systems.

This study makes three key contributions. Firstly, it proposes a socio-technical integration framework that bridges the design-reality gap by shifting from the mere documentation of indigenous signs to the systematic formalisation and validation of LIK through a three-stage pathway: Acquisition, validation and integration. Secondly, it introduces a formalised metadata schema as a design artefact that structures LIK into Attribute, Object, Value, Effect and Action anticipation components. This demonstrates how tacit narratives can be transformed into machine-readable logic units; for example, transforming the observation of ‘cattle restlessness’ into a structured logic unit for tsunami prediction (e.g. Attribute: Animal, Object: Cattle, Value: Restless, Effect: Tsunami and Action anticipation: Run to higher ground). Thirdly, this study delivers an empirical validation method grounded in community consensus. By applying a trust index based on seven validated parameters, we move beyond subjective expert interpretation to provide a computational foundation for disaster warning systems that balances technical precision with human-centred relevance. While the technical implementation of Stage 3 (LIK Integration) is reserved for future research, this study delivers a community-validated knowledge base that is computationally ready for system instantiation.

## Literature review

### Design-reality gap and human factors in Information and communication technology for development

This subsection reviews key perspectives from Information and Communication Technology for Development (ICT4D), a field examining how digital tools are introduced and sustained in developing countries, to explain why technology adoption in rural communities often faces significant contextual challenges. The persistent failure of technology-based interventions in rural areas is rarely a result of technical deficiency; rather, it stems from a design-reality gap that overlooks the human aspect of system interaction (Heeks 2018). Within ICT4D research, this gap occurs when implemented systems are designed based on urban or institutional assumptions that mismatch the localised decision-making realities of rural users. Successful implementation requires contextual integration, where technical success is defined by how effectively the technology couples with existing community behaviour. This gap is further widened by human factors, specifically how individual characteristics shape risk perception and technology trust (Walsham 2017). In rural coastal settings, the adoption of an EWS might be influenced by the recipient’s behavioural profile, including age, fishing experience and their specific role within the fleet. The theoretical foundation of the design-reality gap identifies seven dimensions of distance between system design and local reality: Information, technology, processes, objectives, staffing, management and other resources (Heeks 2018). In rural disaster management, this gap is most prominent in the information dimensions. A centralised design often assumes standardised information quality.

While governments and agencies increasingly deploy ICT-enabled warning platforms, rural users often underutilise them because of limited contextual fit (Baudoin et al. 2016). Disaster warnings that do not match local environmental understanding are perceived as unclear or irrelevant, resulting in delayed or non-response (Mercer et al. 2012). These findings indicate that the success of ICT interventions in rural contexts is not determined by technology availability alone but requires alignment with local knowledge, cultural practices and information interpretation mechanisms. Therefore, this study argues for context-specific system design that integrates human aspects, including cultural dimensions, alongside technical features (Heeks 2018; Walsham 2017). This argument provides the basis for adopting a human aspect in disaster ICT development, where technology must be designed in relation to local knowledge systems and community practices.

### The smart village paradigm and local innovation

**FIGURE 1:**
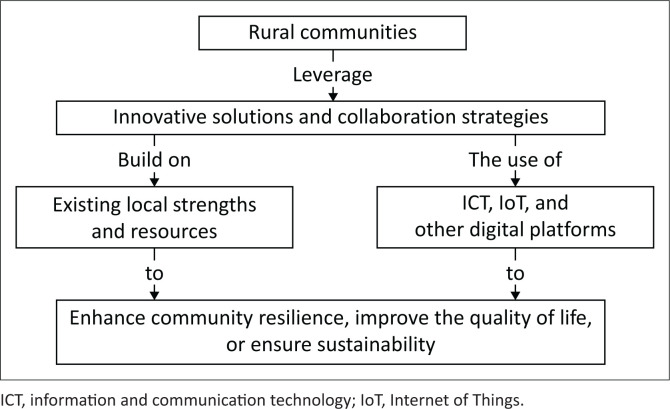
Smart village approach.

The smart village concept offers a foundation for community resilience by empowering communities to utilise local strengths alongside appropriate technology. Rather than replacing traditional practices, this paradigm views technology as an ecosystem that supports bottom-up innovation and community-based problem-solving (Alhari et al. 2022; Mukti et al. 2022; Susanti et al. 2023). This approach marks a critical shift from top-down technological imposition to community-led digital transformation, where rural innovation emerges through the meaningful adaptation of technology to local needs (Dembovska et al. 2023; Li & Zhong 2022; Lombardo, Saeli & Campisi 2023). The concept of Smart Village can be seen in Figure 1.

In top-down approach, the design is often rigid and centralised, leading to interpretability failures in existing national systems. Conversely, the smart village approach positions local knowledge as a strategic development asset, acknowledging that rural communities generate context-specific insights that guide decision-making under environmental uncertainty. This ensures that the system provides the specific recommendations and action-anticipation cues that users currently find lacking in standard platforms.

In this context, LIK is not merely cultural folklore but a form of local innovation that strengthens digital inclusion by ensuring technologies reflect local realities and remain socially acceptable. By prioritising community participation, the smart village approach ensures that disaster governance is co-produced with the people it is intended to protect. This alignment ensures that LIK is not treated as an add-on to disaster technology but is incorporated as a core knowledge component that guides warning interpretation, decision-making and community response.

### Local indigenous knowledge as a decision resource

Local indigenous knowledge refers to context-specific environmental knowledge, practices and interpretations that evolve through long-term interaction between communities and their ecological surroundings according to United Nations Educational, Scientific and Cultural Organization (UNESCO). Transmitted across generations, LIK forms a community-based knowledge system that supports daily survival and disaster recognition. In rural communities with limited access to formal infrastructure, LIK serves as an autonomous early warning mechanism derived from direct environmental sensing (Mercer et al. 2012). Because it is accessible, observable and culturally trusted, LIK plays a significant role in strengthening community resilience. Current research advocates for hybrid strategies where LIK and scientific systems are combined to enhance decision-making (Akanbi & Masinde 2018; Hiwasaki et al. 2014; Limpo et al. 2022; Wang et al. 2019). Despite this potential, LIK remains undervalued in formal disaster management and is often documented as narrative observations rather than actionable knowledge (Hiwasaki et al. 2014). Existing research on LIK–EWS integration remains largely conceptual and fragmented, and most studies focus on documentation rather than demonstrating how knowledge can be systematically embedded into technological architectures. Consequently, LIK often remains external to system design. Two methodological gaps hinder effective integration: Firstly, the validation gap, many efforts rely on expert-driven interpretation of a small number of informants, risking subjective bias. Without broader community validation, integrated LIK may lack community acceptance. This study addresses this by identifying six specific trust parameters of LIK from previous literature and one added parameter found through interview, which are utilisation, continuity, combination, duration, frequency, accuracy and response to quantify community consensus. By measuring these factors, LIK is transitioned from a subjective narrative to objective data points suitable for a trust index. This quantitative grounding ensures that only rules with high community reliability are prioritised for system integration. Secondly, the transformation gap, prior frameworks lack methodological clarity on converting tacit, narrative knowledge into structured, machine-readable formats. Literature rarely offers operational steps for converting LIK into rule-based logic capable of supporting automated early warning decision models. To address these gaps, this study proposes a socio-technical integration framework that treats LIK as a structured knowledge resource. By emphasising a validation phase, the framework ensures LIK is credible, community endorsed and suitable for computational translation. This process serves as a bridge between local wisdom and digital architecture by providing a standardised evidence base for automated decision support.

### Machine learning for knowledge formalisation

A practical implementation gap remains in transforming narrative-based LIK into structured decision rules suitable for digital platforms. Recent research suggests that for LIK to move beyond narrative forms, it must be represented in a format compatible with automated decision models. While this study establishes a trust index through community validation, empirical data indicate that the hierarchical importance of LIK signs is not static, and it fluctuates across different geographical contexts and specific parameters. This dynamic variance presents a complex challenge for traditional, static systems.

Machine learning (ML) is identified as the necessary next-stage pathway for this research to automate the reconciliation between the seven trust parameters and specific behavioural profiles of the community across different geographical contexts. Following methodologies in recent multimodal data behaviour research, Random Forests or Gradients Boosting (XGBoost) can be utilised to identify nonlinear relationships between these parameters. Specifically, by applying SHAP (Shapley Additive exPlanations) values, the model can quantify the feature importance each parameter contributes to safety or unsafe (inaction) responses (Li et al. 2025). This enables the identification of specific parameter combinations that lead to behavioural unsafe (inaction) even when high-accuracy signs are present. By positioning these validated rules as training features for future ML models, the framework provides a computational foundation for hybrid, personalised warning systems. This approach allows for the determination of optimal alert thresholds, calculating whether the interaction between LIK trust parameters and behavioural traits necessitates an extra warning to overcome the risk of unsafe (inaction) behaviour. Consequently, the framework moves beyond simple documentation to become a predictive system for optimising disaster response within the specific coastal fishing communities.

## Research methods and design

This study employs a Design Science Research Methodology (DSRM) to address the design-reality gap in rural disaster management. DSRM is particularly appropriate for ISs research as it focuses on the creation and evaluation of innovative artefacts to solve real-world problems (Peffers et al. 2007). The process begins with problem identification, investigating why national warning systems have low usability and often fail because of a lack of community acceptance. Based on these gaps, the objectives phase defines the requirements for the solution. During design and development, the actual framework is built by transforming qualitative interviews from fishermen into a structured format. This is then demonstrated by converting specific local signs, like changes in animal behaviour or sea patterns, into knowledge rules to show the logic works. The framework moves to evaluation, where a large-scale survey of fishermen measures community trust in LIK. The phases of this study are displayed in Table 1.

**TABLE 1:** Design science research methodology phases (DSRM).

DSRM	Problem identification	Objectives	Design and development	Demonstration	Evaluation
DSRM	Sentiment analysis: evaluating national EWS for gaps in interpretability and information quality Gap analysis: investigating the design-reality gap between top down systems and rural user needs	Requirements definition: Establishing criteria for a machine-readable metadata schema and community trust parameters	LIK acquisition through qualitative inquiry: Conducting 17 in-depth interviews with local fishermen and FGD with 8 community heads to identify core LIK components	LIK formalisation: Transforming tacit narratives interview data into 33 distinct LIK rules using the proposed structural schema	LIK validation: Surveying 438 fishermen to quantify consensus and validate sign reliability across regions
Key Results	Identified gaps: National EWS fail to provide actionable meaning; fewer than three-quarters of rural communities in 3 coastal of Indonesia used ICT tools, while LIK remains universally used	Research objectives: Metadata schema for computable logic Community-based validation to ensure integrated knowledge is endorsed by communities	Proposed Artifact: LIK metadata schema (Attributes, Objects, Values, Effects, Action Anticipation) LIK trust parameters (Utilisation, Continuity, Combination, Duration, Frequency, Accuracy, and Response)	Rule-based prototype: A standardised library of community-endorsed LIK signs for digital integration	Empirical validation: confirmed that the reliability of LIK signs is context-dependent based on the trust parameters

*Source:* Adapted from Peffers, K., Tuunanen, T., Rothenberger, M.A. & Chatterjee, S., 2007, ‘A design science research methodology for information systems research’, *Journal of Management Information Systems* 24(3), 45–77. https://doi.org/10.2753/MIS0742-1222240302

DSRM, design science research methodology; ICT, information and communication technology; LIK, local indigenous knowledge; EWS, early warning systems; FGD, focus group discussion.

### Problem identification

The primary motivation for this study is the design-reality gap in rural disaster management, where top-down EWS fail to achieve long-term adoption because of a lack of contextual alignment. Sentiment analysis of national EWS platforms revealed pervasive concerns regarding information quality, specifically citing data delays, forecast inaccuracies and a lack of actionable recommendations.

The initial inquiry confirmed a significant geographic variance in technology utility. In Aceh, formal ICT-based adoption was measured at 70.8%, yet the perceived ICT benefit was notably lower at 60.4%, indicating that nearly 10% of users found the technology ineffective for their needs. In Yogyakarta, while ICT adoption and perceived benefit both reached 100.0%, deeper analysis through the System Usability Scale (SUS) and User Experience Questionnaire (UEQ) revealed that this high usage masks significant frustration. Results identified marginal usability (SUS < 68) and negative dependability. In contrast, LIK remains universally trusted (100% adoption) across all regions. The identified problem is the lack of an interpretive bridge that can successfully translate technical signals into the social logic required for community action.

### Objectives for solution

The objective of this research is to develop a socio-technical integration framework that enables the systematic transformation of tacit LIK into a formalised, machine-readable format. The solution must meet two primary criteria: (1) it must provide a structured metadata schema capable of transforming narrative LIK into computable logic, and (2) it must establish a community-based validation to ensure integrated knowledge is endorsed by the communities. To achieve this, the solution identifies seven specific trust parameters: Utilisation, continuity, combination, duration, frequency, accuracy and response. By meeting these objectives, it provides a computational foundation for future hybrid EWS that integrate technology with indigenous environmental sensing.

### Design and development

This stage focuses on the creation of the LIK metadata schema, a design artefact developed through thematic analysis of qualitative narratives. The study was conducted in three rural coastal regions of Indonesia characterised by high exposure to hydrometeorological hazards and strong LIK traditions: Pangandaran (West Java), Lhoknga (Aceh) and Depok (D.I. Yogyakarta). These locations were selected based on their direct exposure to the Indian Ocean and historical experiences with major disasters, such as the 2004 and 2006 tsunamis. Table 2 shows the details of study location and the different characteristics it possessed.

**TABLE 2:** The study location.

Location	Geographical setting	Interaction level with environment	Type of communities
Depok, D.I Yogyakarta	Facing Hindia Ocean.	Moderate risk of coastal erosion because of high wave. No historical tsunami, but potential exists.	Moderate-seasonal, limited fleet (679 fishermen)
Pangandaran, West Java	Facing Hindia Ocean. Located on a depositional coastline within a bay.	High tsunami risk, proven by the 2006 tsunami disaster. High wave action and shoreline erosion.	High-large fishing community (1672 fishermen)
Lhoknga, Aceh	Facing Hindia Ocean.	Very high tsunami risk was devastated in 2004 (over 20 m wave run-up). Coastal erosion, tectonic activity.	High-large and structured fishing community (3474 fishermen)

Data acquisition involved 17 in-depth interviews with local fishermen to elicit detailed narratives of environmental sensing and hazard interpretation. This was followed by a focus group discussion (FGD) with eight heads of fishermen communities to validate and refine the emerging insights, ensuring the data reflected shared community consensus rather than isolated observations. The resulting schema structures LIK into five distinct components: Attribute, Object, Value, Effect and Action anticipation. The core innovation of this artefact is the action anticipation, which captures the specific community-endorsed protective measures triggered by an environmental sign.

### Demonstration and evaluation

The artefact’s (LIK metadata schema) were evaluated through a quantitative process. Firstly, the acquisition and formalisation method was demonstrated by converting raw qualitative interview transcripts into 33 distinct LIK. Secondly, the artefact was subjected to an empirical evaluation through a survey of 438 fishermen to quantify community consensus. To ensure evaluation rigour, we implemented an operationalisation protocol where participants anchored responses in core memories of high-stake disaster events to mitigate recall bias. The reliability of the validation instrument was confirmed via a test–retest protocol with 20 fishermen, yielding an intraclass correlation coefficient (ICC) > 0.8, which indicates high temporal stability in the community’s assessment of sign reliability and behavioural triggers.

### Ethical considerations

Ethical clearance to conduct this study was obtained from the Gadjah Mada University Medical and Health Research Ethics Committee under request number (Ref. No. 882609/UN1/FTK.2/DTETI/KM/2025).

## Results

The findings presented in this section serve as the empirical validation of the proposed socio-technical artefacts. Rather than merely documenting LIK, the results quantify the actionable utility and context sensitivity of the formalised rules, providing a data-driven mandate for their integration into automated EWS logic.

### Quantifying the design-reality gap

This study initially addressed the problem identification phase of the DSRM by quantifying the gap between existing technical solutions and community needs. The survey results provided confirmation of the design-reality gap. A sentiment analysis of general Indonesian users of national EWS platforms like InfoBMKG revealed pervasive concerns regarding information quality, specifically citing data update delays, forecast inaccuracies and lack of interpretability where technical alerts failed to provide clear, actionable meaning for the user. While these sentiments reflect a broad national user base, they signify a critical information gap for rural communities that require local, real-time data for appropriate decision-making. While LIK adoption was found to be universal, with 100% of fishermen confirming its use and perceived benefit, the adoption of formal ICT-based disaster systems was significantly lower.

**FIGURE 2:**
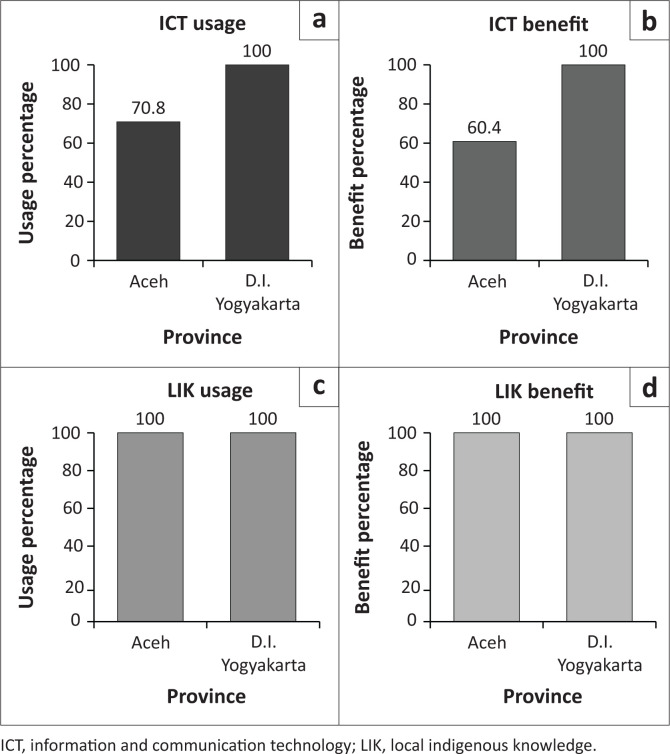
Information and communication technology vs local indigenous knowledge usage and benefit.

Furthermore, empirical data from 438 fishermen from coastal fishing communities reveal that this gap is compounded by a significant geographic divide in technological adoption. While ICT usage reached 100.0% in D.I Yogyakarta, it dropped to 70.8% in Aceh, illustrating that the technology dimension of the gap is highly context dependent (Figure 2). Critically, even where adoption is present, the perceived benefit of ICT is notably lower at 60.4% in Aceh.

While the survey indicated a 100.0% ICT adoption and benefit rate in D.I Yogyakarta, a deeper investigation was conducted to determine whether this high usage masked underlying frustrations. Further surveys utilising the SUS and UEQ in the coastal fishing communities of D.I Yogyakarta region revealed more data regarding the actual quality of interaction. This analysis confirms a significant disconnect, the platform received a SUS score below 68, indicating marginal usability, while UEQ scores for dependability and efficiency were categorised as neutral to negative. These metrics identify a reality where the system exists but fails to achieve its objectives because it provides warning information without the recommendations or action-anticipation cues required for rural decision-making. When these gaps, whether in access, interpretability or actionable information, are not addressed, the result is a partial failure where technology is present but ignored in favour of trusted LIK. These data prove that an integration approach is required to bridge high-trust indigenous logic with under-performing technical infrastructure.

### Artefact design and development: Local indigenous knowledge metadata schema

To enable the future integration of LIK into technology-based EWS, the knowledge must be transformed from narrative descriptions into a structured, machine-readable format. Field analysis reveals that the LIK used by fishermen is not unstructured; rather, it follows a consistent internal logic where each observation contains core components explaining what is detected, how it is interpreted and what it implies for safety.

To address RQ1, this subsection demonstrates how the developed metadata schema formalises tacit indigenous narratives into structured logic. The schema was developed through a two-stage process involving a literature review followed by a qualitative analysis of 17 in-depth interviews with local fishermen and a FGD with eight heads of fishing communities.

**FIGURE 3:**
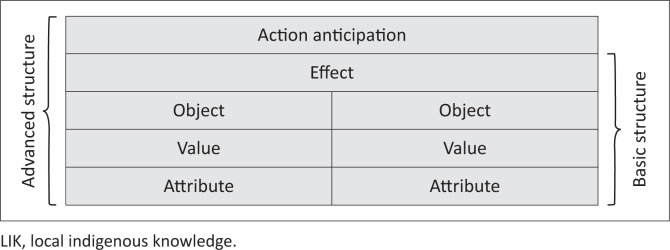
Local indigenous knowledge metadata schema.

This iterative process identified five essential components required to bridge oral tradition with computable logic. The LIK metadata schema is displayed in Figure 3.

While the initial literature review provided a basic structure for environmental observation, it was the qualitative field inquiry that revealed the necessity of a second, more advanced structure. Specifically, the interviews and FGDs highlighted that for LIK to be actionable, it must include action anticipation, the community-endorsed safety response triggered by a sign.

These components allow LIK to be represented in a form that follows logical reasoning, making it compatible with knowledge bases, decision rules and ML. The structure can be generalised as:

IF [Object: X] has [Value: A]

AND [Object: Y] has [Value: B]

THEN [Effect: Disaster]

ACTION RECOMMENDATION [Action Anticipation]

For example:

IF [Object: Tide movement] has [Value: Calm]

AND [Object: Water level] has [Value: Sudden recession]

THEN [Effect: Tsunami]

ACTION RECOMMENDATION: [Action Anticipation: stay away from coastline]

In many cases, fishermen emphasised that a single environmental indicator is insufficient to trigger a safe response (taking action anticipation). Instead, they cross-validate multiple LIK signals before making safety decisions. For example, while domestic animals appearing restless or agitated are a known indicator, it is rarely enough on its own to prompt an evacuation to higher ground. It must be accompanied by secondary signs, such as the appearance of sudden, anomalous waves during clear weather. Similarly, a notably calm sea state since morning or experiencing no typical tidal movement is not perceived as an immediate threat until it is corroborated by a sudden recession of the sea level or a felt earthquake. This indicates that LIK frequently operates through multi-condition logic. During analysis, several such combinations were identified across the three study locations, many of which are utilised consistently by all fishing communities. This cross-regional consistency suggests a strong collective validation of compound LIK rules. From a design perspective, this structured representation enables the systematic conversion of narrative local knowledge into rule-based logic. These validated, multi-factor rules provide the necessary foundation to be encoded into ML models for EWS, acting as the essential computational bridge between raw indigenous observations and automated disaster logic.

### Artefact demonstration: From narrative to logic rules

To systematically identify and structure LIK into a machine-readable format, the framework utilises a process called LIK acquisition. This stage focuses on identifying indigenous knowledge through expert consultation, including 17 in-depth interviews with local fishermen and a FGD with eight heads of fishing communities. The acquired data are then organised into a format that allows it to be processed and utilised by digital systems. This acquisition phase is divided into two distinct sub-stages: LIK identification and LIK formalisation. Local indigenous knowledge identification involves documenting and classifying various forms of local knowledge, such as animal behaviours and environmental cues, through the involvement of domain experts. As shown in Table 4, tacit narratives like ‘A sudden recession of the sea level’ or an ‘Pets and Domestic animals appear restless (or agitated)’ are extracted from interview transcripts and assigned specific codes (e.g. Ts-3 to Ts-6) based on their geographical relevance in West Java, Aceh or Yogyakarta. This LIK identification example can be seen in Table 4.

**TABLE 3:** Local indigenous knowledge metadata schema explanation.

Component	Meaning	Question it answers	Example
Attribute	General element of nature being observed	What part of nature is being observed?	Wind, waves, sky, birds, clouds
Object	Specific part of the attribute	What exactly is being observed from that element?	Wind direction, wave interval, cloud colour
Value	Condition or state measured/observed	What condition is happening?	Strong, sudden change, dark, rising
Effect	Meaning or predicted hazard linked to the sign	What does this sign indicate?	Extreme waves, strong winds, tsunami
Action anticipation (optional)	Response taken by fishermen	What action should be done?	Be cautious, adjust fishing time, stay away from the coastline

LIK, local indigenous knowledge.

**TABLE 4:** Local indigenous knowledge identification.

Transcript interview	LIK code	LIK identification	Interview location			Interview sources	FGD
			West Java	Aceh	Yogyakarta		
That morning, the seawater looked calm, experiencing neither high nor low tide. Just before the tsunami occurred, the seawater began to recede suddenly, then a roaring sound was heard, it was an earthquake. After that, the seawater immediately rose quickly and massively. Initially, we were just observing the surroundings while being cautious; after the earthquake, we ran away from the beach.	Ts-1	The seawater has been calm since morning (experiencing no high tide or low tide)	Yes	No	No	Interview-2	Yes
	Ts-2	A rumbling sound accompanied by an earthquake	Yes	Yes	No	Interview-2, 5, 7, 8 and 9	Yes
	Ts-3	A sudden recession of the sea level	Yes	Yes	No	Interview-2, 3, 7, 8 and 9	Yes
Tsunami is preceded by an extreme recession of sea level, followed by large waves suddenly arriving on land. This event occurs even if the weather is clear, the wind is weak and the earthquake is not felt strongly.	Ts-4	Sudden, very large waves appearing during clear weather	Yes	Yes	No	Interview-5, 6, 7, 8 and 9	Yes
One of the natural signs for a tsunami is when everything suddenly goes completely silent. That morning, there was no sound from the birds. The fish were not jumping. The wind was not blowing, and you could not even see a squirrel moving. The whole environment just felt completely still. The only sounds you could hear were man-made things, like cars or motorcycles. It was an eerie, heavy silence.	Ts-5	The environment is extremely quiet and still, occurring during clear weather	No	Yes	No	Interview-10	Yes
Starting the night before the tsunami, we noticed the cattle and the chickens; they were just restless, really agitated and not right. And the dogs all disappeared from the village, as if they had already moved or migrated somewhere else. Even the animals that were kept in cages were showing clear signs of distress.	Ts-6	Pets and domestic animals appear restless (or agitated)	No	Yes	No	Interview-10	Yes

LIK, local indigenous knowledge; FGD, focus group discussion; Ts, Tsunami.

Local indigenous knowledge formalisation subsequently structures this identified knowledge into a structured format following LIK metadata schema. Table 3 presents the five-component logic (Attribute, Object, Value, Effect, and Action anticipation), which is demonstrated through the narrative transformations shown in Table 5. For instance, a narrative about a sudden recession (Ts-3) is formalised into specific variables where the ‘Attribute’ is Sea, ‘Object’ is Water Level, ‘Value’ is Sudden Recession, ‘Effect’ is Tsunami and the ‘Action anticipation’ is to stay away from the coastline. This structured representation provides the necessary bridge between narrative LIK and computable disaster logic.

**TABLE 5:** Local indigenous knowledge formalisation.

LIK code	LIK identification	Attribute	Object	Value	Effect	Action anticipation	Type of LIK	Prediction type
Ts-1	The seawater has been calm since morning (experiencing no high tide or low tide)	Sea	Tidal movement	Calm (no high or low tide)	Tsunami	Be cautious	Environmental cues	Disaster warning (tsunami prediction)
Ts-2	A rumbling sound accompanied by an earthquake	Atmosphere	Ambient sound	Rumbling	Tsunami	Stay away from the coastline	Environmental cues	Disaster warning (tsunami prediction)
		Seismic	Ground sensation	Felt earthquake				
Ts-3	A sudden recession of the sea level	Sea	Water level	Sudden recession	Tsunami	Stay away from the coastline	Environmental cues	Disaster warning (tsunami prediction)
Ts-4	Sudden, very large waves appearing during clear weather	Sea	Wave condition	Very large	Tsunami	Run to the higher ground	Environmental cues	Disaster warning (tsunami prediction)
		Atmosphere	Weather condition	Clear				
Ts-5	The environment is extremely quiet and still, occurring during clear weather	Atmosphere	Ambient sound	Extreme silence	Tsunami	Be cautious	Environmental cues	Disaster warning (tsunami prediction)
		Atmosphere	Weather condition	Clear				
Ts-6	Pets and domestic animals appear restless (or agitated)	Animal behaviour	Pets	Distressed	Tsunami	Be cautious	Animal behaviour	Disaster warning (tsunami prediction)

LIK, local indigenous knowledge.

### Empirical evaluation of the artefact: Local indigenous knowledge community validation

**FIGURE 4:**
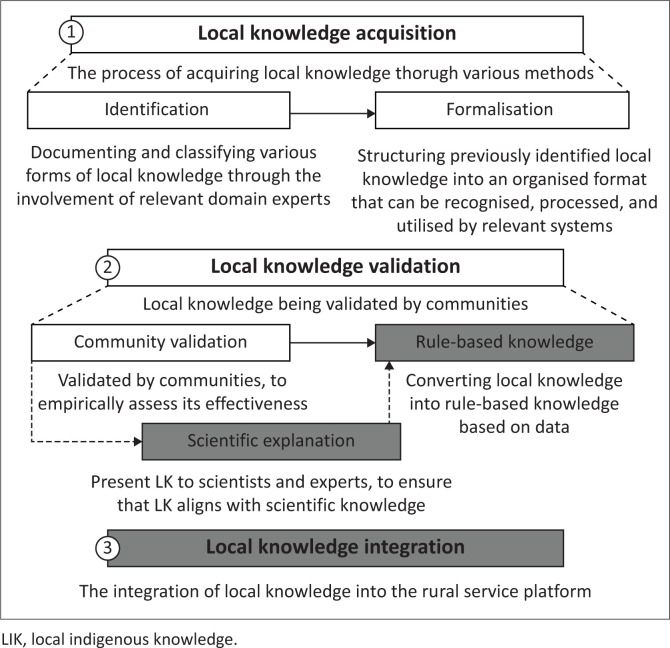
Local indigenous knowledge acquisition and local indigenous knowledge validation stage.

Following the evaluation phase of the DSRM, the formalised rules were subjected to a community-consensus test to answer RQ2. To transition from expert-driven identification to a data-driven representation of the broader population, the LIK validation stage was conducted through a survey of 438 fishermen across the study locations. This stage verifies the identified indigenous knowledge through community consensus, moving beyond subjective interpretation, while the other stages such as rule-based knowledge and LIK integration are reserved for further research. The entire stages can be seen in Figure 4: LIK acquisition and LIK validation stage.

By applying a trust index to measure reliability, we calculated mean scores across seven trust parameters found from the literature review and interview: Utilisation, Continuity, Duration, Frequency, Accuracy, Response and Combination. Table 6 details the definition of each parameter and the questions that were asked during the survey.

**TABLE 6:** Local indigenous knowledge trust index parameters.

Parameters	Definition	Questions used in survey
Utilisation	The number of the fisherman applying traditional indicators (natural signs or animal behaviour) rooted in LIK for the detection or prediction of tsunami events.	Have you ever used those natural signs or animal behaviour to predict a tsunami?
Continuity	The current and consistent use of traditional indicators (natural signs or animal behaviour) for tsunami detection by community members in their present-day activities and observations.	Are those natural signs or animal behaviour still being used today to predict a tsunami?
Duration	The quantifiable period (e.g. years) that a fisherman or the community has actively utilised traditional LIK indicators (natural signs or animal behaviour) for tsunami event forecasting.	How long have you been using those natural signs or animal behaviour to predict a tsunami?
Frequency	The quantifiable measure of recurrence (e.g. daily, weekly or monthly) of the practice of observing LIK indicators (natural signs or animal behaviour) for the purpose of tsunami detection.	How often do you make observations of those natural signs or animal behaviour?
Accuracy	The calculated ratio or percentage that compares the total number of correct tsunami predictions/warnings (where a tsunami actually occurred following the LIK warning) against the total number of times the LIK indicators (natural signs/animal behaviour) were observed and interpreted for a potential tsunami event.	What is the percentage of tsunami occurrences compared to the number of predictions?
Response	The specific type and sequence of protective behaviours or communication activities initiated by the respondent directly following the observation of LIK indicators associated with tsunami prediction.	What action do you take when you see those natural signs or animal behaviour?
Combination	The complement LIK signs used to trigger specific response.	Any other natural signs or animal behaviour used to trigger another level of response?

LIK, local indigenous knowledge.

**FIGURE 5:**
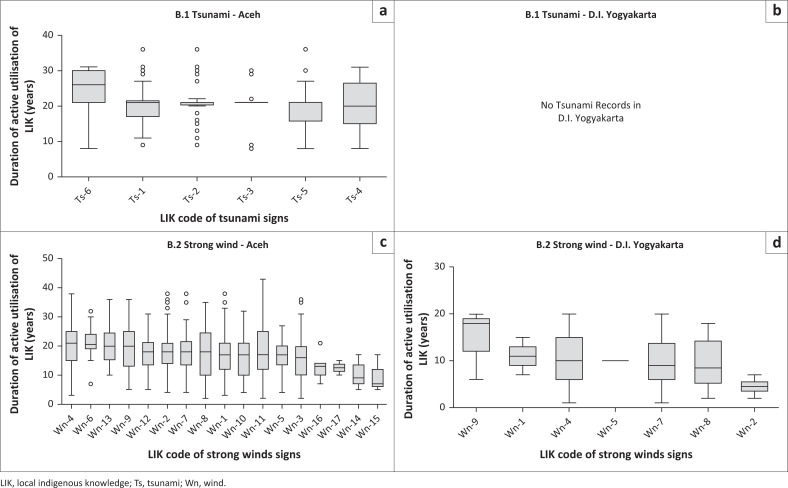
Trusted local indigenous knowledge signs order based on parameter duration.

**FIGURE 6:**
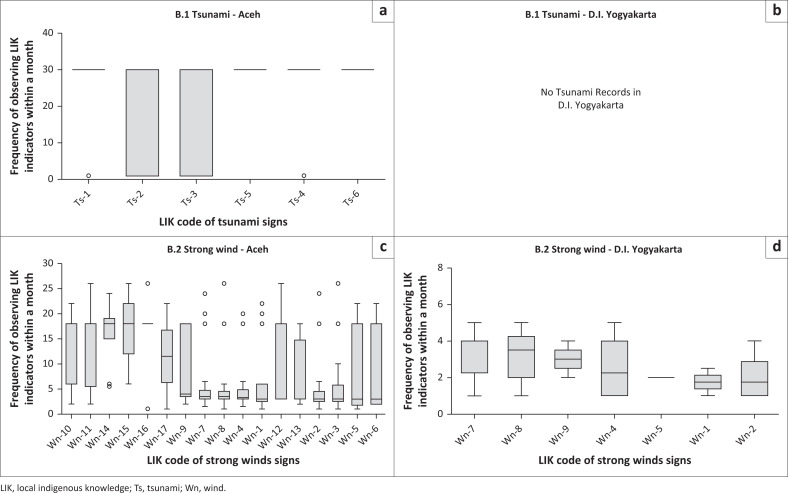
Trusted local indigenous knowledge signs order based on parameter frequency.

**FIGURE 7:**
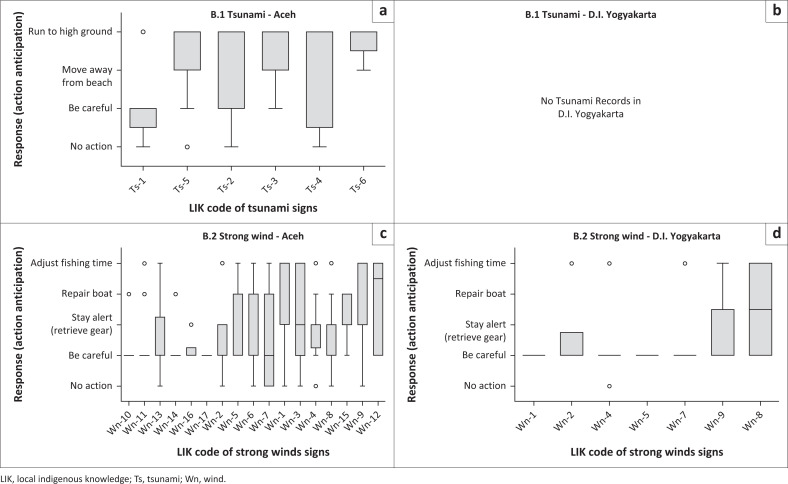
Trusted local indigenous knowledge signs order based on parameter response.

**FIGURE 8:**
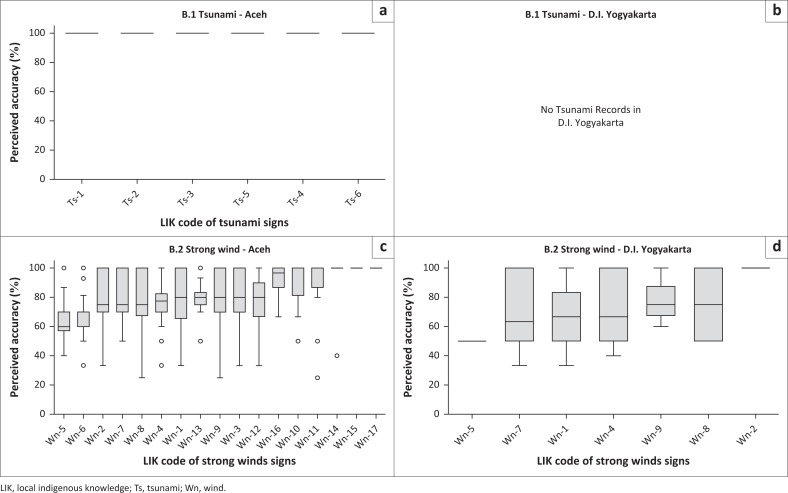
Trusted local indigenous knowledge signs order based on parameter accuracy.

The results demonstrated that these seven parameters significantly re-order the hierarchy of LIK signs beyond simple accuracy. In terms of duration (Figure 5), Ts-6 (Pet Distressed) was ranked higher than Ts-4 (Sudden, anomalous waves), suggesting it is perceived as a more persistent signal. However, the order reversed when evaluating frequency (Figure 6) and response (Figure 7), where Ts-4 was ranked higher; this indicates that while Ts-6 is more persistent, Ts-4 is more effective at actually triggering protective actions. These shifts suggest that even though both indicators were universally validated with 100% accuracy (Figure 8), their perceived reliability varies significantly across different trust dimensions.

This quantitative validation proves that reliability is not a single-variable metric; instead, it converts raw observations into rule-based knowledge. By identifying which signs are most reliable across specific parameters, the framework provides the necessary evidence to determine which LIK rules are trusted enough for automated system integration and which require additional cross-validation.

### Contextual sensitivity and behavioural profiling

Another significant finding of this evaluation is the impact of geographic settings and behavioural variance on LIK trust. While tsunami-related rules (Ts-1 to Ts-6) maintained universal accuracy across all sites, weather-related LIK signs such as Wn-1 (descending clouds) showed regional variance, with 91.2% accuracy rating in Aceh compared to 80.4% in Yogyakarta. This variance confirms that the framework serves as a diagnostic tool to pinpoint critical, context-specific rules for different coastal regions rather than relying on a generalised model.

Furthermore, the data suggest that actionable utility (defined by the parameter of response) is significantly influenced by the fisherman’s behavioural profile. Key factors include age, fishing experience, fishing role, fishing location, number of experiences with different types of disasters and number of interactions with disasters. These factors dictate response readiness. For example, the data identified a specific overconfident profile, typically characterised by older fishermen with extensive sea experience, who may ignore high-accuracy LIK signs.

**FIGURE 9:**
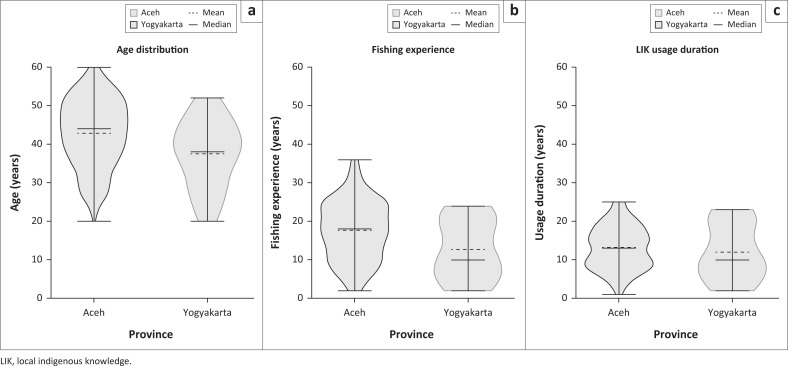
Behavioural profile (age, fishing experience) based on study location.

The regional findings highlight that the study locations possess distinct behavioural profiles, which further influence the varying levels of trust in LIK signs. As illustrated in Figure 9, the comparative data for Aceh and Yogyakarta, factors such as age distribution, fishing experience and LIK usage duration show clear differences between the two fishing communities.

**FIGURE 10:**
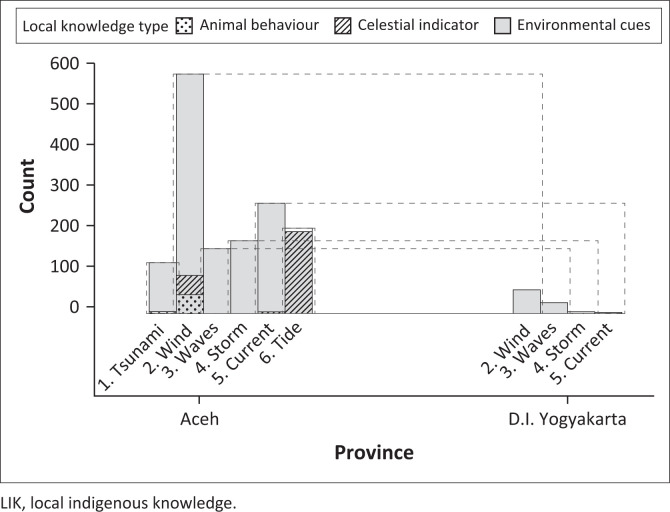
Type of local indigenous knowledge signs trusted based on the behavioural profile of the fishing location.

Specifically, the Aceh profile is characterised by a higher mean age and more extensive fishing experience compared to D.I. Yogyakarta. These differences in experience and age contribute directly to how each community prioritises LIK parameters and responds to environmental triggers. Furthermore, the fishing location significantly dictates the specific types of LIK utilised by each community. For instance, fishing communities in Aceh often operate in locations far from the shore, requiring them to stay at sea for several weeks. Because they must observe signs while in the middle of the sea, often at night, they rely more heavily on celestial indicators rather than animal behaviours or coastal environmental cues that are difficult to monitor in deep-water or nighttime settings. Figure 10 shows that the celestial indicator only appears in Aceh.

Because of these distinct behavioural landscapes, the selected study locations serve as a significant choice for testing the framework. They allow for a comparative analysis of how socio-demographic backgrounds shape the reliability and actionable utility of LIK. These findings lay the groundwork for future research involving ML. By integrating the weighted trust index with these specific behavioural profiles, a multi-dimensional decision matrix can be developed. This matrix will enable future EWS to provide customised notifications, determining whether to issue a standard warning or an extra warning based on the interaction between the sign’s reliability and the unique behavioural characteristics of the recipient.

## Discussion

The findings presented in ‘result’ section are not merely a collection of statistical data; they represent the demonstrated utility of the DSRM artefact. This study transforms a narrative, tacit knowledge into a structured and quantitatively validated knowledge base. This discussion interprets these results by analysing the framework’s role in bridging the validation gap and outlining its theoretical and practical implications. The problem identified in the literature is the persistent validation gap, which leads to EWS misalignment. This study provides a solution by replacing subjective expert interpretation with community-centric validation. The scores generated through the survey are not an external interpretation of reliability; they are the community’s own perceived reliability, quantified through seven trust parameters: Utilisation, continuity, duration, frequency, accuracy, response and combination. This establishes a bottom-up mandate for each LIK rule, creating a level of credibility that expert-driven models alone cannot achieve. From a theoretical perspective, this research provides a mechanism for quantitative participation in the system’s core logic, moving beyond participation as a simple buzzword. Practically, the validation scores serve as a design blueprint for human-centred EWS, allowing designers to prioritise critical alerts based on confirmed community trust. Several limitations must be acknowledged. Firstly, the geographic scope was restricted to Indonesian coastal communities along the Indian Ocean. While the framework is sensitive to regional variations, the specific rules identified are context-specific to maritime environments. Secondly, the potential for recall bias in community narratives was addressed by anchoring the survey in core memories of life-threatening events and utilising a test–retest protocol (ICC > 0.8) to ensure temporal stability. Thirdly, while the LIK metadata schema formalises the LIK structure, the synergy of multiple cues suggests that static rules alone may not capture the full complexity of human interpretation. This justifies identification of ML as the next essential stage for handling weighted, multi-condition reasoning and integrating behavioural profiles.

## Conclusion

This study addresses the design-reality gap in rural disaster management by proposing and evaluating a socio-technical integration framework for the acquisition and validation of LIK for rural EWS. By transforming tacit observations into a formalised LIK metadata schema (Attribute, Object, Value, Effect and Action anticipation components), this research provides a computational foundation that enables indigenous wisdom to be integrated into technological EWS. The primary contribution of this research is the transition from mere documentation to a validation method based on community consensus and empirical trust parameters. This approach ensures that LIK acts as an interpretive bridge, aligning technical alerts with the cognitive models and decision-making logic of rural communities. While this article focuses on the design and evaluation of the underlying logic, it delivers a community-validated knowledge base that is computationally ready for technical integration. For policymakers and practitioners, the study demonstrates that successful disaster governance depends on human-centred alignment rather than top-down implementation. Integrating validation processes that respect both scientific and LIK enhances system trust and response readiness. Future research will focus on the Stage 3 (LIK integration) artefact, utilising ML to optimise the interaction between the trust index and behavioural profiles. Pilot-testing this logic within live digital platforms will further strengthen the resilience of rural coastal communities.
